# Green synthesis of V_2_C MXene quantum dots with tunable nonlinear absorption for optical limiting applications

**DOI:** 10.1039/d5na00777a

**Published:** 2025-10-07

**Authors:** Amrutha Shivappanayaka, Hasana Jahan Elamkulavan, Vari Sivaji Reddy, Chandrasekharan Keloth

**Affiliations:** a Laser and Nonlinear Optics Laboratory, Department of Physics, National Institute of Technology Calicut-673601 India csk@nitc.ac.in

## Abstract

Vanadium carbide MXene quantum dots (V_2_C QDs) have emerged as promising nanoscale materials with tunable surface chemistry and pronounced quantum confinement effects. Herein, we report a green, HF-free synthesis of highly fluorescent V_2_C QDs directly from the MAX phase *via* pulsed laser ablation in a binary solvent system. The synthesized quantum dots exhibited a production yield of 27% and a narrow size distribution, with an average diameter of 2.5 nm. Furthermore, they exhibit intense and stable photoluminescence with a quantum yield of 11.5%. Their optically tunable emission, combined with excellent optical stability, positions them as strong candidates for high-resolution bioimaging and biosensing applications. We investigated their nonlinear optical response using an open-aperture Z-scan technique at 532 nm, which revealed a dual behavior, namely saturable absorption at low excitation intensities and strong reverse saturable absorption at higher intensities. These materials also show a good optical limiting performance, characterized by a low onset threshold. The unique coexistence of stable fluorescence and robust nonlinear optical properties makes V_2_C QDs an attractive option for advancements in laser protection, optoelectronics, and multifunctional biomedical photonics. These results provide a sustainable approach to synthesizing high-quality V_2_C QDs and highlight their potential in bridging nanophotonics and biomedicine through versatile optical functionalities.

## Abstract 2

Environmentally friendly synthesized V_2_C quantum dots exhibit strong fluorescence and intensity-dependent nonlinear absorption, making them promising candidates for optical limiting and multifunctional photonic devices.

## Graphical abstract

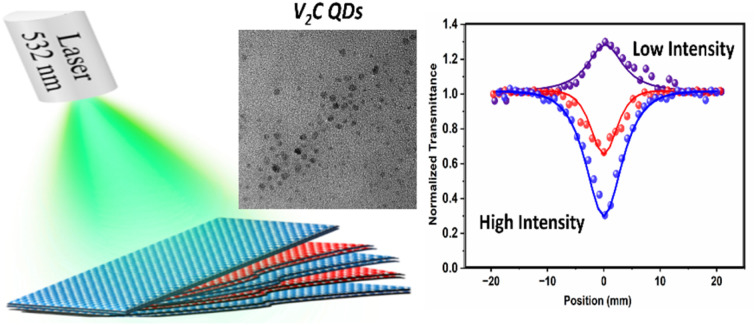

## Introduction

MXenes are two-dimensional (2D) materials that have emerged as a transformative class of nanostructures, exhibiting a unique combination of atomic-scale thickness, tunable electronic structures, and high surface reactivity.^1,2^ These features endow them with remarkable potential for integration into diverse technological domains, including biosensing, biomedical engineering, energy conversion, catalysis, and environmental remediation.^3,4^ In particular, their exceptional optical and electronic tunability has led to growing interest in their use in nonlinear optics and photonic platforms.^5^ Their ability to support strong light–matter interactions makes them promising candidates for next-generation optoelectronic devices, including photodetectors, optical modulators, terahertz components, and saturable absorbers in ultrafast photonics.^6,7^ MXene materials are derived from layered ternary carbides and nitrides, commonly referred to as MAX phases.^8–11^ These MAX phases are composed of a transition metal (denoted as M), an element that is typically from group 13 or 14 (denoted as A), and carbon and/or nitrogen (denoted as X). The transformation of MAX phases into MXenes involves the selective removal of the A-layer, resulting in structures with the general formula M_*n*+1_X_*n*_T_*x*_, where T_*x*_ refers to surface terminations such as –O, –OH, and –F.^12–14^

In the space where nanomaterials intersect with the ever-evolving world of optics, MXene quantum dots have gained significant attention.^15^ These ultrasmall, zero-dimensional materials, which are typically less than 10 nm in size, are derived from their two-dimensional MXene counterparts and retain the intrinsic characteristics of MXenes, including strong hydrophilicity and versatile surface tunability.^16–18^ In addition to their inherent properties, these quantum dots exhibit unique optical and electronic behavior that is strongly size-dependent, primarily due to the quantum confinement effect. Due to these promising properties, QDs have a strong potential for advanced applications in nonlinear optics, optoelectronics, sensing, and bioimaging.^19–22^

One pioneering method that is widely used for synthesizing QDs with enhanced optical properties is etching the MAX phase with hydrofluoric acid (HF), followed by hydrothermal treatment.^23,24^ To address the hazards associated with HF, alternative fluoride-free techniques were developed. For example, a method utilizing tetrabutylammonium hydroxide (TBAOH) for the selective removal of Al layers, combined with ultrasonication, allowed the preparation of fluorine-free QDs from MAX precursors.^14,25–27^ However, HF etching often introduces structural defects, dangling bonds, and residual fluoride functional groups, which degrade the photoluminescence properties, making QDs less suitable for optoelectronic applications.^28^ Furthermore, HF is highly toxic, corrosive, and difficult to handle safely, making it hazardous for laboratory researchers and industrial-scale applications.^29^ These limitations have prompted the scientific community to explore HF-free strategies that are safer, faster, and more environmentally friendly.

One such promising alternative is pulsed laser irradiation in liquids (PLIL).^30^ The primary benefits of PLIL are its ability to produce high-purity, ligand-free QDs without the need for hazardous chemical precursors, making it particularly suitable for applications in bioimaging and optoelectronics.^30,31^ A recent study has reported on green, HF-free laser ablation in water for synthesizing QDs and their applications in areas such as photocatalysis^32,33^ and lasing.^34^

Recent advances in nonlinear optical limiting materials have significantly expanded the landscape of photonic protection, with Ti_3_C_2_T_*x*_/polymer hybrids, perovskite quantum dots, and plasmonic MXene composites emerging as leading candidates under nanosecond laser excitation.^35^ Covalently functionalized Ti_3_C_2_T_*x*_ hybrids, such as porphyrin-linked MXenes, have demonstrated nonlinear absorption coefficients (*β*_eff_) up to 251.68 cm GW^−1^ and low optical limiting thresholds (0.62 J cm^−2^), attributed to synergistic two-photon absorption (TPA), excited-state absorption (ESA), and efficient interfacial charge transfer. Perovskite quantum dots (*e.g.*, CsPbBr_3_ and MAPbBr_3_) embedded in polymer matrices exhibit strong reverse saturable absorption (RSA) and onset thresholds in the range of 0.52–0.63 J cm^−2^, while plasmonic MXene composites, especially those incorporating gold or silver nanoparticles, leverage localized surface plasmon resonance and hot carrier effects to further enhance the nonlinear response and broadband limiting performance.^35–37^

Recent studies on transition metal molybdates, such as erbium-doped Ag_22_MoO_44_, have shown that defect engineering and rare-earth doping can further boost the NLO efficiency, with two-photon absorption coefficients increasing from 0.85 × 10^−10^ m W^−1^ (pure) to 6.22 × 10^−10^ m W^−1^ (0.5% Er-doped), and a marked reduction in the optical limiting threshold.^38,39^ Nonlinear optical materials are fundamental to the progress of contemporary photonic technologies, providing the essential properties required for devices such as optical limiters, switches, and communication systems.^40^ V_2_C MXene, in particular, has attracted interest for its strong light–matter interactions and narrow bandgap, making it a promising candidate for nonlinear photonics.^41^ V_2_C nanosheets and hybrids have been explored in mode locking applications due to their promising NLO properties at 1.9 μm.^41,42^ However, there are no reports demonstrating the optical limiting behavior in V_2_C QDs, especially those synthesized *via* the PLIL method.

To the best of our knowledge, this is the first report on the optical limiting capabilities of V_2_C QDs synthesized using a green, ethanol–water-assisted pulsed laser ablation method. This binary solvent plays a crucial role in facilitating efficient exfoliation and high yield in a short interval of time. This eco-friendly approach not only eliminates the need for hazardous chemicals, but also facilitates the scalable production of high-quality, surface-functionalized V_2_C QDs that exhibit excitation-dependent emission and a quantum yield of 11.5% without chemical passivation. Nonlinear optical properties of synthesized QDs were studied using the z-scan technique, which reveals tunable absorption and demonstrates robust optical limiting performance with low onset and threshold values. Our experimental findings not only advance the green synthesis of MXene quantum dots, but also present V_2_C QDs as promising candidates for next-generation nonlinear optical, laser safety, bioimaging, and optoelectronic technologies.

## Material synthesis

**Fig. 1:**
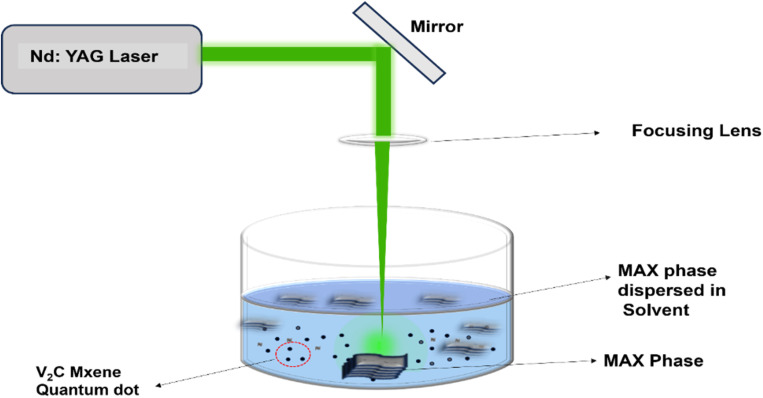
Schematic of the pulsed laser ablation set-up for the synthesis of V_2_C QDs.

V_2_C QDs were synthesized from the V_2_AlC MAX phase (Nanochemzone, Canada) using the PLIL technique.^43^ In a typical synthesis, 5 mg of high-purity V_2_AlC MAX phase powder (≥98%) was dispersed in 5 mL of a 1 : 1 ethanol-deionized water mixture. The dispersion was ultrasonicated for 30 minutes to ensure a homogeneous distribution of the MAX phase particles before transferring it to a 10 mL glass beaker. A Q-switched Nd: YAG laser, operating at a 532 nm wavelength with a 7 ns pulse duration, a 10 Hz repetition rate, and 40 mJ pulse energy, served as the excitation source. The beam was focused at the centre of the dispersion using a plano-convex lens with a 10 cm focal length. Laser irradiation was conducted for 15 minutes under ambient conditions. The synthesis of V_2_C QDs has been significantly advanced through the adoption of pulsed laser ablation in a binary ethanol–water solvent system, which enhances both synthesis efficiency and purity of QDs. The unique physicochemical interplay between ethanol and water reduces the surface tension and viscosity, extends the lifetime of the cavitation bubbles, and facilitates the dynamic surface modification.^44^ This process generates intense localized plasma and shockwaves at the focal point, initiating the rapid exfoliation of the V_2_AlC MAX phase into few-layered V_2_C nanosheets.^33^ Then, further irradiation for 15 minutes produced the V_2_C QDs. The solution was filtered through a 0.45 μm syringe filter to remove larger aggregates and unexfoliated material. The filtrate underwent a two-step centrifugation process: an initial spin at 4000 rpm for 30 minutes to remove the remaining debris, followed by a high-speed spin at 13 000 rpm for 1 hour to isolate the well-dispersed V_2_C QDs. The final supernatant, containing rich and stable fluorescent V_2_C QDs, was then subjected to rotary evaporation to efficiently remove the solvent. Then, we calculated the V_2_C QDs production yield, which was found to be 27% (production yield refers to the mass yield, calculated as the ratio of the mass of isolated V_2_C QDs to the mass of the initial V_2_AlC precursor used in the synthesis process). Starting from 5.00 mg of V_2_AlC precursor, we obtained 1.35 mg of dried V_2_C QDs. This yield is substantially higher and more time-efficient than reported techniques using pure water.^33,45^Fig. 1 displays the schematic illustration of the V_2_C QDs synthesis procedure.

## Characterization

An extensive set of analytical techniques was employed to thoroughly investigate the structural, morphological, and optical characteristics of the synthesized V_2_C QDs and MAX phase. X-ray diffraction (XRD) measurements were carried out using a Rigaku Smart Lab SE diffractometer equipped with Cu Kα radiation (*λ* = 1.5406 Å). The morphological profile and elemental composition of the samples were examined using field-emission scanning electron microscopy (FESEM) and energy-dispersive X-ray spectroscopy (EDX) on a Carl Zeiss Sigma 300 system. Further confirmation of the formation of V_2_C QDs was achieved through high-resolution transmission electron microscopy (HRTEM) using a JEOL JEM-2100 instrument. We have performed functional group analysis using a PerkinElmer UATR Two Fourier transform infrared (FTIR) spectrometer. Optical absorption behavior was investigated using a Shimadzu UV-2450 UV-Vis spectrophotometer. Steady-state fluorescence, quantum yield (QY), and photoluminescence (PL) lifetime measurements of the V_2_C QDs were carried out using a Horiba FluoroMax-4 spectrofluorometer. The open-aperture (OA) z-scan technique was utilized to study the nonlinear absorption properties. A Q-switched Nd:YAG laser (Quanta Ray, Spectra Physics) operating at 532 nm wavelength, 7 ns pulse width at 10 Hz repetition rate was used as an excitation source. X-ray photoelectron spectroscopy (XPS) was performed to analyze the elemental composition and chemical states of the synthesized material using a monochromatic Al Kα X-ray source (*hν* = 1486.6 eV).

## Results and discussion

### Properties of the bulk vanadium carbide MAX phase (V_2_AlC)

**Fig. 2:**
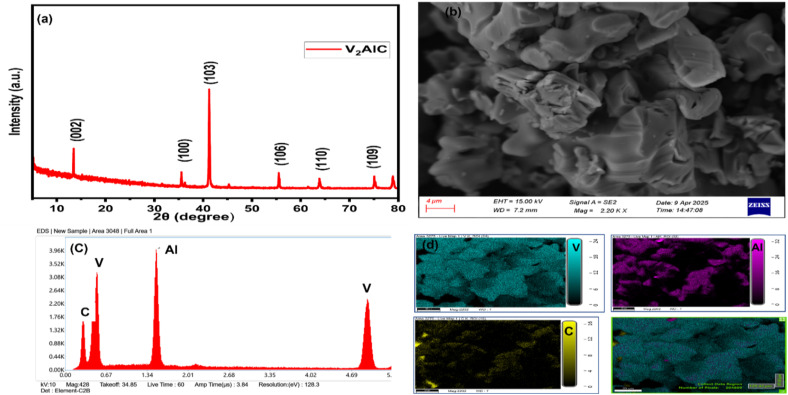
(a) XRD pattern, (b) FE-SEM micrograph, (c) EDS spectrum, and (d) EDS elemental mapping of the bulk V_2_AlC MAX phase.

Fig. 2(a) illustrates the XRD pattern of the V_2_AlC MAX phase. The diffraction peaks align with the standard reference pattern JCPDS No. 29-0101, confirming the hexagonal crystal structure.^46^ Distinct reflections at 2*θ* ≈ 13.0°, 36.0°, 41.8°, 55.0°, and 60.0° correspond to the (002), (100), (103), (106), and (110) planes, respectively, validating the phase identity. The absence of impurity peaks indicates high phase purity, while the sharp and intense peaks reflect the material's high crystallinity.^47,48^ The FESEM image of the V_2_AlC powder shown in Fig. 2(b) reveals a layered and densely packed structure typical of the MAX phase. This structure is advantageous for further delamination or processing into MXenes. The EDS analysis, presented in Fig. 2(c), confirms the presence of vanadium (V), aluminium (Al), and carbon (C). Additionally, the elemental mapping shown in Fig. 2(d) illustrates a homogeneous distribution of these elements across the sample, supporting the compositional uniformity.^41^ These characterizations affirm that the purchased V_2_AlC MAX phase exhibits high purity, excellent crystallinity, and a typical layered morphology, making it an ideal precursor for preparing V_2_C QDS.

### Properties of the synthesized V_2_C QDs

**Fig. 3:**
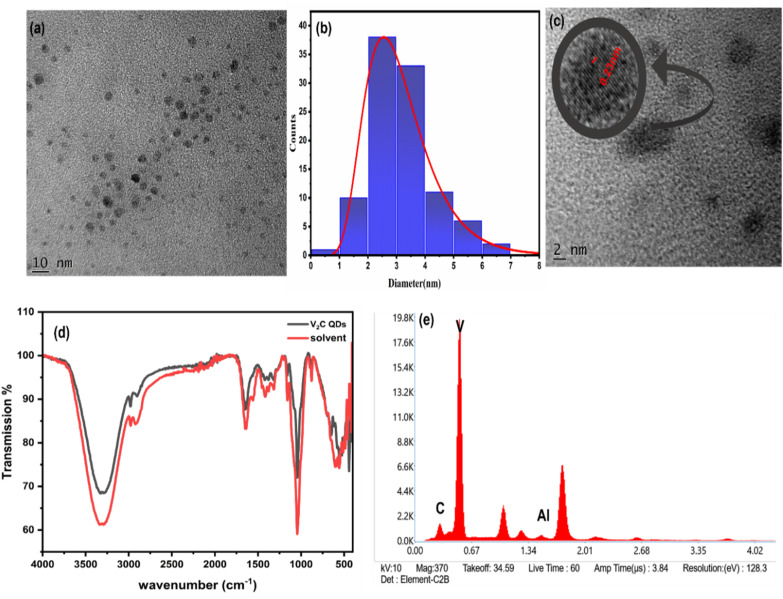
(a) HRTEM image of the V_2_C QDs produced by the PLIL method. (b) Size distribution histogram of the V_2_C QDs. (c) Magnified HRTEM image showing the presence of lattice fringes. (d) FTIR spectrum of the V_2_C QDs dispersed in an ethanol and water mixture. (e) EDS spectra.

The HR-TEM image shown in Fig. 3(a) confirms the successful formation of V_2_C QDs. The corresponding size distribution histogram presented in Fig. 3(b) indicates a narrow distribution, with an average particle size of approximately 2.5 nm. This uniform and ultrasmall size significantly enhances the possibility of quantum confinement effects, a characteristic typically found in quantum dots. The zoomed-in image of the individual quantum dot, shown in Fig. 3(c), exhibits well-defined lattice fringes, confirming its high crystallinity at the nanoscale. The measured interplanar spacing is approximately 0.23 nm, which corresponds to the (100) plane of the V_2_C Mxene.^49–52^ The presence of such lattice features validates that the MXene-derived quantum dots retain their core crystalline phase even after pulsed laser fragmentation, indicating structural robustness and phase purity. The synthesized V_2_C QDs exhibited a zeta potential of −23.2 mV, indicating good colloidal stability. The negative zeta potential value observed for the V_2_C QDs suggests that sufficient surface charge prevents particle aggregation, thereby maintaining uniform dispersion. To investigate the surface chemistry of the synthesized V_2_C QDs, FTIR spectroscopy was performed. The FTIR spectrum of V_2_C QDs shown in Fig. 3(d) reveals several characteristic vibrational features that confirm the presence of surface functional groups. Absorption bands in the 500–900 cm^−1^ range are associated with V–C and V–O vibrations, providing clear evidence of the preserved vanadium carbide structure at the nanoscale. A broad band between 3200 and 3500 cm^−1^ corresponds to the O–H stretching vibrations of hydroxyl groups, which are probably introduced during laser irradiation in the ethanol–water medium.^53,54^Fig. 3(e) presents the EDS spectrum of V_2_C QDs, revealing a clear elemental composition. Dominant peaks corresponding to vanadium (V) and carbon (C) confirm the formation of V_2_C QDs, while the near absence of the (Al) peak indicates the effective removal of Al from the V_2_AlC MAX phase precursor. This underscores the efficiency of the pulsed laser ablation in liquid method, particularly in an ethanol–water binary solvent system, in the selective removal of Al. The absence of any additional peaks other than a substrate further affirms the high purity of the synthesized QDs, validating the PLIL process as a reliable route for producing phase-pure V_2_C QDs.

**Fig. 4:**
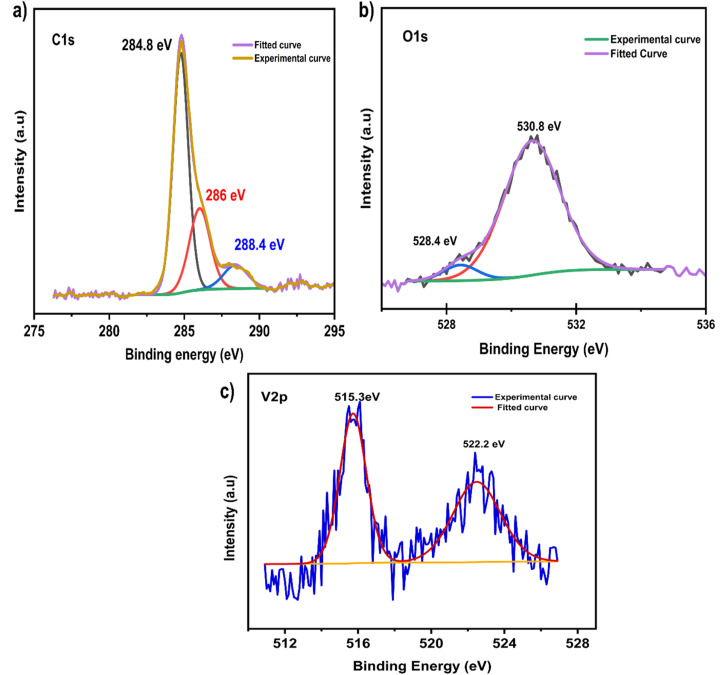
XPS spectra of (a) C 1s, (b) O 1s, and (c) V 2p of the V_2_C QDs synthesized *via* PLIL method.

Fig. 4 presents the XPS analysis of V_2_C QDs synthesized through laser ablation in binary solvents, providing detailed insight into their surface chemistry, elemental composition, and purity, which are crucial for understanding their functional properties. The deconvoluted C 1s spectrum shown in Fig. 4(a) exhibits a dominant peak at 284.8 eV corresponding to C–C and V–C bonds, indicating the carbide framework, alongside smaller peaks at approximately 286 eV and 288.4 eV assigned to C–O/C–OH and C=O groups, respectively.^33,55^ These oxygen-containing functionalities result from the solvent environment during ablation, which facilitates functional group attachment, enhancing the surface hydrophilicity and dispersibility of the quantum dots. Fig. 4(b), which shows the O 1s spectrum, further supports this by showing peaks at 530.8 eV and 528.4 eV, attributed to lattice oxygen and surface metal–oxygen bonds, respectively.^56^

The V2p spectra in Fig. 4(c) display characteristic peaks at 515.3 eV (V 2p_3/2_) and 522.2 eV (V 2p_1/2_). This is consistent with vanadium in lower oxidation states typical of V_2_C MXene, confirming that there is no V_2_O_5_ formation or contamination, which further supports the integrity of the carbide phase after synthesis.^49^ Overall, these XPS results convincingly demonstrate that the laser ablation method in ethanol not only preserves the core MXene structure of the V_2_C quantum dots, but also introduces beneficial oxygen-containing functional groups such as –OH, –O, and –COOH on their surface.

**Fig. 5:**
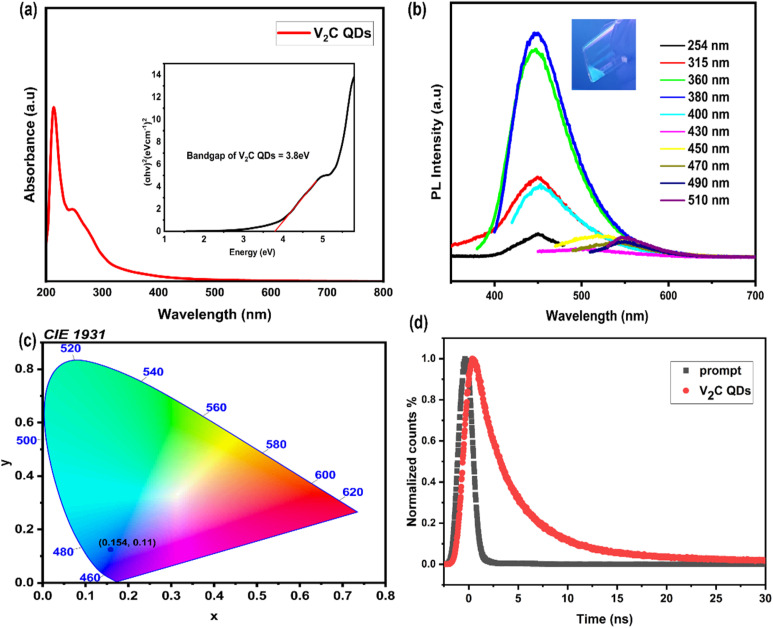
(a) UV-Vis absorption spectrum of the V_2_C dispersed in a 1 : 1 ethanol and water mixture (the inset corresponds to the Tauc plot for optical bandgap estimation). (b) Photoluminescence spectra at different excitation wavelengths, with the inset showing a photograph of the V_2_C QDs solution under UV light. (c) The CIE chromaticity diagram for an excitation of 380 nm. (d) The fluorescence decay curve of the synthesized V_2_C QDs and the prompt.

The UV-Vis absorption spectra of V_2_C QDs shown in Fig. 5(a) contain a sharp absorption peak at approximately 220 nm, a secondary shoulder peak at 270 nm, and a weak absorption is observed in the entire visible region. In the inset of Fig. 4(a), the Tauc plot yields an estimated bandgap of approximately 3.8 eV.

The photoluminescence spectra of V_2_C QDs shown in Fig. 5(b) demonstrate tunable multicolour emission. As the excitation wavelength increases from 254 to 510 nm, the emission peak gradually shifts toward longer wavelengths. For example, excitation at 254 nm gives an emission peak at 447 nm, while excitation at 470 nm shifts it to around 550 nm. The strongest and brightest emission is observed at 447 nm when the QDs are excited at 370 nm, producing a strong blue fluorescence. The tunable fluorescence emission of V_2_C QDs is likely a result of the quantum confinement effect, and the presence of diverse surface functional groups and defects may introduce multiple surface energy states that serve as possible alternative sites for exciton recombination, which could result in excitation-dependent emission. Consequently, the emission colour of V_2_C QDs can probably be tuned by adjusting both the size of the quantum dots and their surface chemistry. The inset in Fig. 5(b) displays a photograph of the QDs dispersed in solvent under UV illumination, visually confirming the strong blue fluorescence. The synthesized V_2_C QDs exhibited an absolute quantum yield of 11.5% for an excitation wavelength of 370 nm. The quantum yield was measured by using the integrated sphere calibration. The PL excitation and emission plots are provided in the SI.

The CIE 1931 *xy* chromaticity diagram is a standardized graphical representation of all colors perceptible to the human eye, based on human visual response developed by the International Commission on Illumination (CIE). It serves as a foundational model for quantifying and comparing colors in a two-dimensional space, where the *x* and *y* coordinates correspond to chromaticity values derived from the CIE 1931 color matching functions.^57^ Each point within the diagram corresponds to a specific hue and saturation, independent of brightness. The color purity of an LED can be determined by its position on the chromaticity diagram, based on its *x* and *y* coordinates. For the V_2_C QDs prepared in this study, the measured chromaticity values were (*x*, *y*) = (0.154, 0.11), as shown in Fig. 5(c). These results show that V_2_C QDs have great potential for use in optical devices. In particular, they could be good candidates for making blue LEDs.

The fluorescence lifetime of the as-synthesized V_2_C QDs was measured using the time-correlated single photon counting technique (Fig. 5(d)). The fluorescence decay profile of the V_2_C QDs was best fit by a 3-exponential model, suggesting the involvement of three distinct decay pathways. The corresponding time constants (*T*) and relative amplitudes (*A*) for each decay process are provided in Table 1. The mean fluorescence lifetime was calculated using eqn (1), which is given by yielding a value of *τ* = 7.8 ns at an excitation wavelength of 370 nm.

1 *τ* = *A*_1_*T*_1_ + *A*_2_*T*_2_ + *A*_3_*T*_3_

**Table 1:** Time constant and relative amplitude obtained from the fluorescence decay fitting of the V_2_C QDs

S. no.	Time constant, *T* (ns)	Norm. amplitude, *a*
1	3.585	0.654
2	0.422	0.078
3	20.13	0.271

### Nonlinear absorption studies

**Fig. 6:**
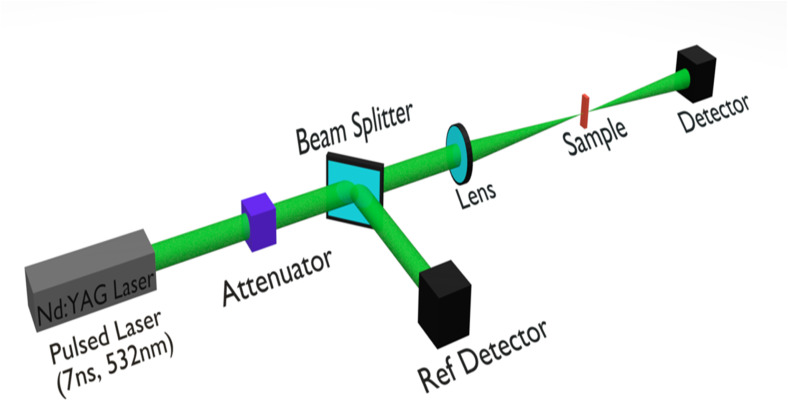
Schematic representation of the open-aperture z-scan set-up.

The open-aperture z-scan technique was employed to investigate the nonlinear absorption behavior of the sample. A schematic of the experimental setup is illustrated in Fig. 6.

In this configuration, a laser beam is focused along the propagation axis (*z*-direction) using a convex lens with a focal length of 15 cm. The sample, contained in a thick quartz cuvette, is mounted on a motorized translation stage that enables precise movement along the *z*-axis in 1 mm increments through the focal region. As the sample traverses the path of the focused Gaussian laser beam, its transmittance at varying incident intensities is monitored using a pyroelectric detector coupled with an energy ratio meter. This setup allows for the measurement of the sample's transmission characteristics as a function of position and, consequently, laser intensity of the focused beams, which fulfills the thin sample approximation required for accurate z-scan analysis. The beam waist in our setup was measured to be 18.5 μm, with a Rayleigh range of approximately 2 mm, ensuring a proper Gaussian beam profile during the open-aperture Z-scan experiments focused on nonlinear absorption. Importantly, control measurements performed on the pure solvent under the same excitation energies and conditions showed no detectable nonlinear absorption response at these intensities. The reproducibility of the measurements was meticulously verified to ensure that the focused laser did not cause sample degradation. Specifically, each measurement was conducted at a slightly different location within the quartz cuvette, and the laser power was maintained below the sample's damage threshold.

**Fig. 7:**
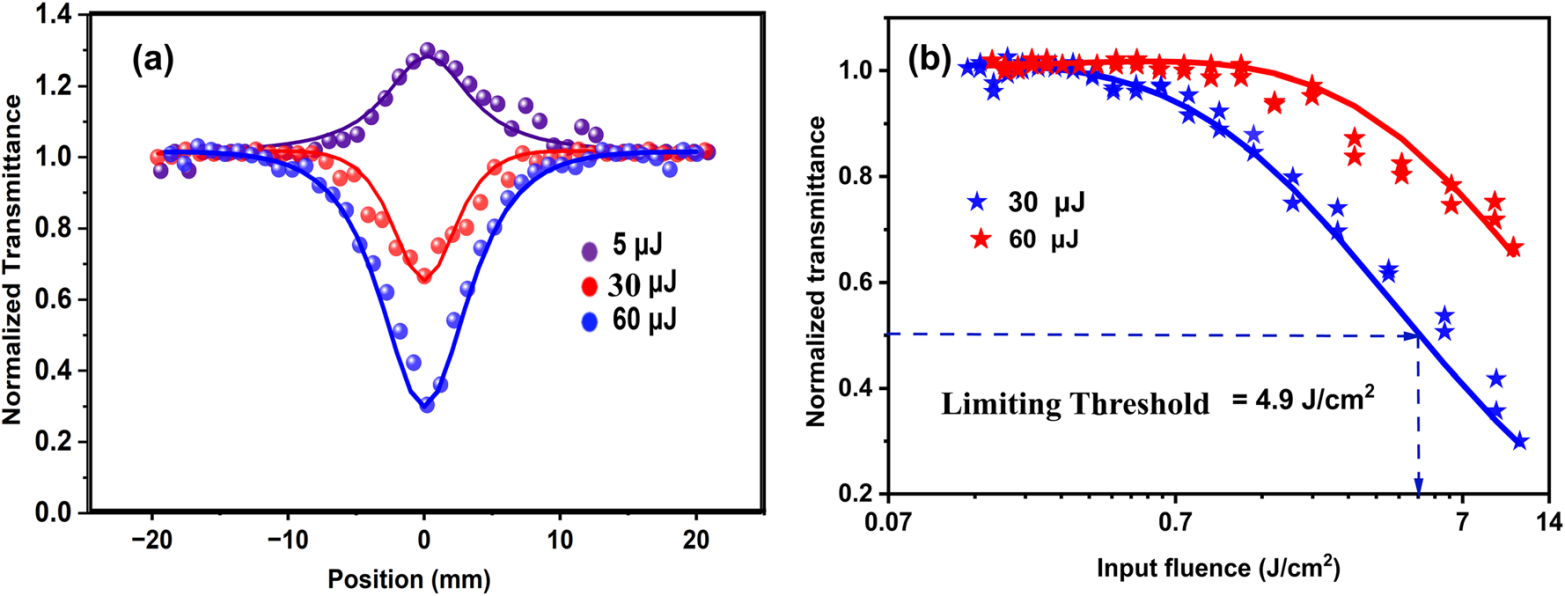
(a) OA z-scan signatures of the V_2_C QDs at various on-axis beam intensities: 0.14 GW cm^−2^ (5 μJ), 0.84 GW cm^−2^ (30 μJ), and 1.68 GW cm^−2^ (60 μJ). (b) Optical limiting plot.

Fig. 7(a) shows the OA z-scan plots at different on-axis intensities, 0.14 GW cm^−2^ to 1.68 GW cm^−2^. In this curve, the normalized transmittance is defined as the ratio of the sample's transmittance at each position along the *z*-axis to the transmittance measured when the sample is positioned far from the focal point. This allows for changes in transmittance relative to the focal plane to be represented as a function of the sample's position (*z*). At lower intensities, the plot exhibits a clear peak at the focal point (*z* = 0), indicating saturable absorption (SA). In this regime, the material becomes less absorbent as the light intensity increases, permitting more light to pass through. As the intensity gradually increases, a noticeable dip begins to form at the focus, which becomes deeper with further increases. At the highest intensities, only the dip remains, signifying a shift to reverse saturable absorption (RSA), where the material absorbs more light as the intensity rises, resulting in decreased transmission at the focal point. The solid line shown in the figure represents the theoretical model that best fits the experimental data. To accurately model this nonlinear optical response, the nonlinear pulse propagation equation (eqn (2)) is applied. This framework allows for the extraction of essential parameters such as the saturable intensity (*I*_s_), a characteristic of saturable absorption, and the effective nonlinear coefficient (*β*_eff_), which encapsulates the combined contributions from both 2 PA and ESA.^58^2
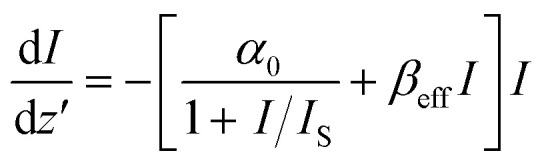
Here, *I* refers to the intensity of the incident laser beam, d*I*/d*z*′ represents its rate of change along the propagation axis (*z*), and *α*_0_ is the linear absorption coefficient. The first term is responsible for the SA, and the second term governs the RSA. Table 2 presents the retrieved values of the saturation intensity and the effective nonlinear absorption coefficient for various excitation intensities. At the lowest input energy, a distinct saturable absorption response was observed. Under these conditions, the experimental data were best described with *β*_eff_, indicating minimal nonlinear absorption at low excitation levels.

**Table 2:** Variation of the saturation intensity and effective nonlinear absorption coefficient of the sample concerning different input laser intensities along the beam axis

On-axis intensity (GW cm^−2^)	*I*_s_ (10^−2^ GW cm^−2^)	*β*_eff_ (cm GW^−1^)
0.14	0.04	0
0.84	0.319	8
1.68	0.059	19

**Fig. 8:**
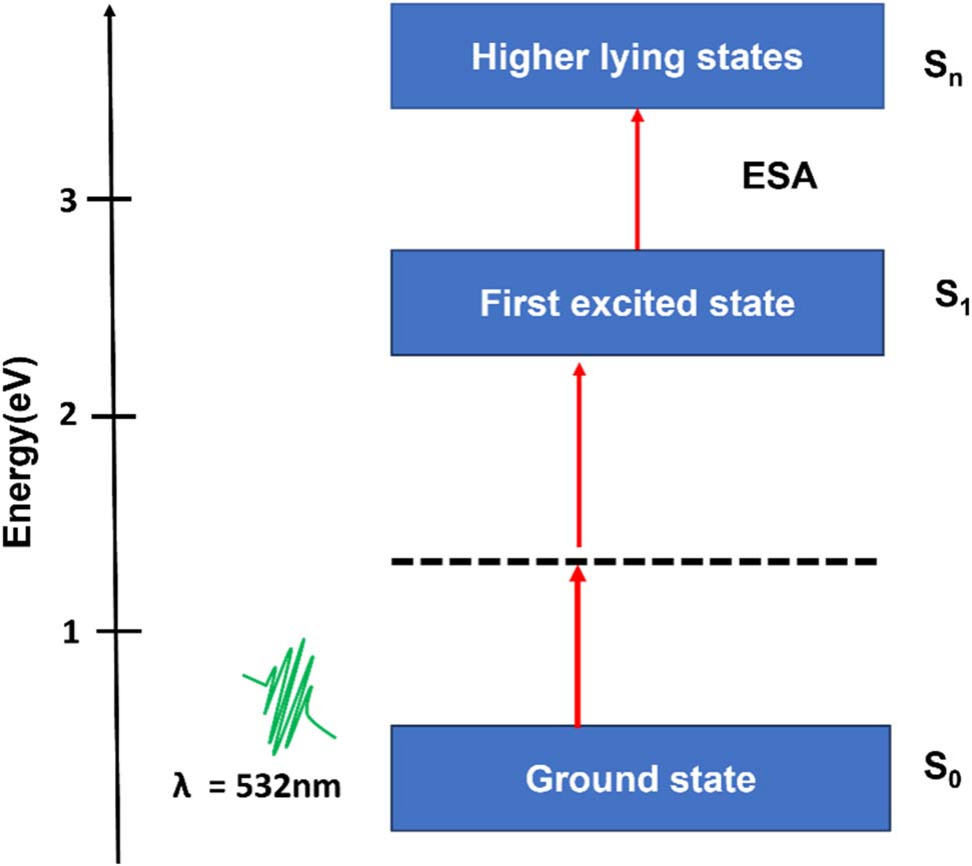
Schematic of the NLO absorption mechanism of the V_2_C QDs.

This RSA behavior is may be attributed to the combined influence of ESA and weak TPA. To justify the reason for the RSA at higher input intensities, we have a simple three-level energy diagram as shown in Fig. 8, where S_0_ is the ground state, S_1_ is the lowest lying first excited state, and *S*_*n*_ is the higher excited state. At low excitation intensities, the observed SA behavior may be primarily attributed to the presence of surface functional groups, predominantly hydroxyl (OH) termination introduced during the PLIL synthesis. These terminations generate localized surface or band-tail states within the bandgap, near the conduction and valence band edges.^59^ As a result, although the intrinsic bandgap of V_2_C QDs is 3.8 eV, limited sub-bandgap absorption at 532 nm (2.33 eV) becomes feasible through these surface states. With increasing intensity, the available states become saturated, leading to a decrease in absorption, a characteristic hallmark of saturable absorption.^60^ At higher excitation intensities, the absorption mechanism transitions to RSA, dominated by nonlinear optical processes. While direct single-photon absorption is energetically forbidden at 532 nm, the simultaneous absorption of two photons (4.66 eV) enables excitation from the ground state to the higher state S_1_*via* a weak TPA process. Once electrons populate the S_1_ state, further photon absorption promotes them to *S*_*n*_*via* ESA. As the excitation intensity increases, ESA plays a more prominent role.^60^ To quantitatively validate this mechanism, the excited state absorption cross-section (*σ*_e_) and ground state absorption cross-section (*σ*_g_) were determined using the relations,^61^
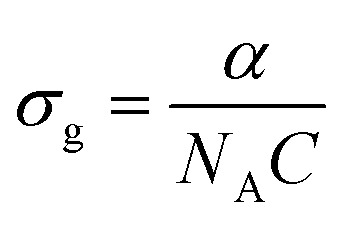
 and 
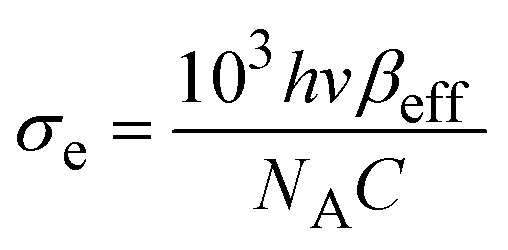
, where *α* = 1.9 cm^−1^ is the linear absorption coefficient, *h* is Planck's constant, *v* is the frequency, *β*_eff_ = 1.9 × 10^−8^ cm W^−1^ is the effective nonlinear absorption coefficient, *C* = 7.2 μmol cm^−3^ is the concentration, and *N*_A_ is Avogadro's number. Based on these calculations, the values of *σ*_e_ and *σ*_g_ were found to be 46.41 × 10^−19^ cm^2^ and 4.67 × 10^−19^ cm^2^, respectively. The fact that *σ*_e_ is nearly ten times greater than *σ*_g_ provides strong evidence for the ESA-dominated RSA behavior at high excitation intensities.^61^ This SA-to-RSA transition may be due to the interplay between surface states and nonlinear optical processes in quantum-confined systems. The nonlinear optical properties of V_2_C QDs with other V_2_C topographies, such as nanosheets, show more pronounced NLO effects due to their reduced dimensionality and enhanced quantum confinement, which leads to discrete energy states and higher nonlinear absorption cross-sections. Nanosheets of V_2_C, while offering larger surface area and easier film formation, often have weaker NLO responses because their electronic states are more delocalized and subject to scattering losses. On the other hand, nanosheets may provide better thermal stability and mechanical robustness for device fabrication.^41,60^ In the case of the V_2_C MXene QDs, the combination of quantum confinement, high surface reactivity, and significant excited-state interactions underscores their potential for applications in optical limiting, all-optical switching, and intensity-dependent photonic devices.

### Optical limiting applications

We then investigated the optical limiting performance of the V_2_C QDs using data derived from open-aperture z-scan measurements. An ideal optical limiter functions by allowing high transparency at low incident light intensities, while increasing its absorption as the intensity rises, thereby protecting sensitive optical components from potential damage.^62^ Moreover, such a limiter should possess a low onset threshold, high limiting efficiency, and a large damage threshold.^63^ As shown in Fig. 7(b), the V_2_C QDs exhibit clear optical limiting behavior, with an onset threshold of 0.29 J cm^−2^ and a limiting threshold of 4.9 J cm^−2^, indicating a strong nonlinear absorption response under nanosecond laser excitation at 532 nm. These values are comparable to or better than several previously reported nanomaterials (as summarized in Table 3), highlighting the excellent potential of V_2_C QDs for laser protection. Given their sustainable, chemical-free synthesis, high optical nonlinearity, and broadband transparency at low intensities, V_2_C QDs can be considered promising candidates for advanced optical limiters. Their robust performance is expected to enhance the safety and efficiency of laser-based systems and optical sensors across various practical platforms.

**Table 3:** Comparison of the optical limiting threshold and limiting onset values of the V_2_C QDs with those of other materials

Sample	Wavelength (nm)	*β*_eff_ (cm GW^−1^)	Limiting threshold (J cm^−2^)	Limiting onset value (J cm^−2^)
V_2_C QDs (present study)	532	19	4.9	0.29
Ti_3_C_2_ nanosheets^64^	532	5.5	—	—
3-(Fluor pyrrolidinium) MnCl_3_ (ref. 35)	532	4.1 × 10^6^	—	0.009
CsPbBr_3_ (QDs)^65^	532	—	6.4	—
WSe_2_	532	—	7.2	0.99
Graphene^66^	532	—	15.15	0.44
C_60_ (ref. 67)	532	—	3.1	—
WS_2_ (ref. 68)	532	—	7.3	1.47
MoS_2_ (ref. 69)	532	—	11.16	1.52
PtS_2_ (ref. 70)	532	1.113	0.420	0.023

## Conclusion

We have established a rapid, eco-friendly, and scalable strategy for synthesizing highly fluorescent V_2_C QDs *via* pulsed laser ablation in an ethanol–water binary solvent system. This innovative approach yields V_2_C QDs with significantly higher efficiency and purity than previous single-solvent ablation methods, owing to synergistic solvent effects that enhance the yield, nanodot uniformity, and photoluminescence. The as-prepared V_2_C QDs exhibit robust, tunable multi-colour fluorescence and outstanding nonlinear optical behavior, including a low optical limiting onset of 0.29 J cm^−2^ and a threshold of 4.9 J cm^−2^. Notably, these QDs display clear intensity-dependent switching between saturable and reverse saturable absorption, enabling strong and tunable optical limiting capabilities. Such properties make them highly attractive for advanced photonic and optoelectronic applications, including laser protection, integrated optical devices, and high-resolution fluorescence bioimaging. Importantly, the green and scalable nature of this synthesis method highlights the value of binary solvent engineering for sustainable nanomaterial production, paving the way for the future integration of MXene quantum dots in next-generation nanotechnologies.

## Conflicts of interest

The authors declare no conflict of interest.

## Supplementary Material

NA-007-D5NA00777A-s001

## Data Availability

The data supporting this study, are available within the article and its supplementary information (SI). Supplementary information: details of sample's emission and excitation spectra (PL) and nonlinear absorption plot of the solvent (Z-scan). See DOI: https://doi.org/10.1039/d5na00777a.
