# Supplementary material for: Green synthesis of V2C MXene quantum dots with tunable nonlinear absorption for optical limiting applications
Source: Nanoscale Adv. 2025 Oct 7;7(23):7694–704. doi: 10.1039/d5na00777a (PMC12529794; doi:10.1039/d5na00777a)

Supplementary Information (SI) for Nanoscale Advances.
This journal is © The Royal Society of Chemistry 2025

The absolute quantum yield was determined using an integrating sphere calibration (liquid sample, quartz cuvette, absorbance set to 0.10) to ensure all emitted photons were collected under direct excitation at 370 nm. Measurements were performed under identical optical geometry and acquisition conditions for both sample and blank (solvent-only reference), with the solvent refractive index matched between runs, thereby eliminating the need for refractive-index correction. The emission and excitation graphs of both samples and the blank are given in Figure 1.

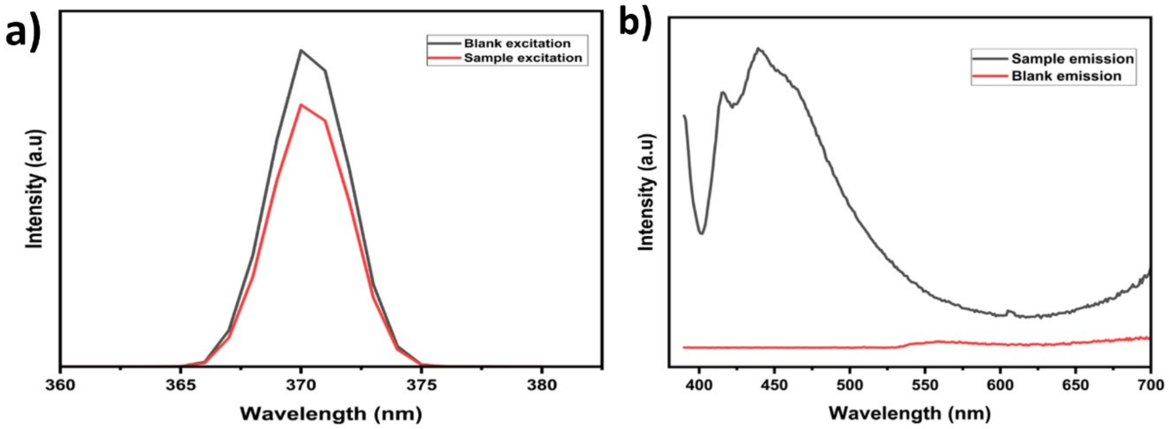

Figure 1. a) Excitation and b) Emission graphs of both sample and blank.

The open-aperture Z-scan technique was employed, and control measurements performed on the pure solvent under the same excitation energies and conditions showed no detectable nonlinear absorption response at these intensities. The solvent NLO profile is given below for 60 μJ.

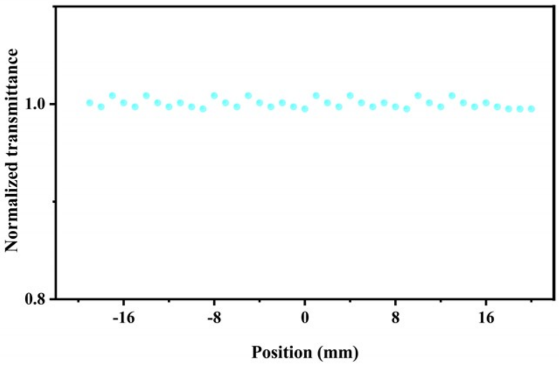

Supplement: NA-007-D5NA00777A-s001 [file NA-007-D5NA00777A-s001.pdf]
